# The effects of socioecological factors on variation of communicable diseases: A multiple-disease study at the national scale of Vietnam

**DOI:** 10.1371/journal.pone.0193246

**Published:** 2018-03-01

**Authors:** Dung Phung, Huong Xuan Nguyen, Huong Lien Thi Nguyen, Anh Mai Luong, Cuong Manh Do, Quang Dai Tran, Cordia Chu

**Affiliations:** 1 Centre for Environment and Population Health, Menzies Health Institute Queensland, Griffith University, Brisbane, Queensland, Australia; 2 Da Nang University of Medical Technology and Pharmacy, Da Nang, Vietnam; 3 Health Environment Management Agency, Ministry of Health, Hanoi, Vietnam; 4 General Department of Preventive Medicine, Ministry of Health, Hanoi, Vietnam; Kansas State University, UNITED STATES

## Abstract

**Objective:**

To examine the effects of socioecological factors on multiple communicable diseases across Vietnam.

**Methods:**

We used the *Moran’s I* tests to evaluate spatial clusters of diseases and applied multilevel negative binomial regression models using the Bayesian framework to analyse the association between socioecological factors and the diseases queried by oral, airborne, vector-borne, and animal transmission diseases.

**Results and significance:**

The study found that oral-transmission diseases were spatially distributed across the country; whereas, the airborne-transmission diseases were more clustered in the Northwest and vector-borne transmission diseases were more clustered in the South. Most of diseases were sensitive with climatic factors. For instance, a 1°C increase in average temperature is significantly associated with 0.4% (95CI, 0.3–0.5), 2.5% (95%CI, 1.4–3.6), 0.9% (95%CI, 0.6–1.4), 1.1% (95%CI), 5% (95%CI, 3-.7.4), 0.4% (95%CI, 0.2–0.7), and 2% (95%CI, 1.5–2.8) increase in risk of diarrhoea, shigellosis, mumps, influenza, dengue, malaria, and rabies respectively. The influences of socio-economic factors on risk of communicable diseases are varied by factors with the biggest influence of population density. The research findings reflect an important implication for the climate change adaptation strategies of health sectors. A development of weather-based early warning systems should be considered to strengthen communicable disease prevention in Vietnam.

## Introduction

It is obvious that rising anthropogenic greenhouse gas emission has led to global warming, which results in more frequently extreme variability in weather factors such as temperatures and rainfalls and have had widespread impacts on human health and ecological system [1,2]. The climate variability is increasing the burden of climate-sensitive health outcomes, including communicable diseases and challenging the ability of health systems in the context of changing climate and development patterns [3]. Many infectious diseases are highly sensitive to climate variation due to influences of changes in weather conditions on the pathogens, vectors, hosts and their living environment [4–7]. Studies revealed that long-term climate warming tends to facilitate the geographic expansion of some infectious diseases [8,9], and extreme weather events may increase opportunities for more clustered disease outbreaks and outbreaks at a non-traditional places and time [10]. The burden of climate-sensitive diseases is predicted to be greatest in developing countries where have tropical climate and low levels of socioeconomic development and coverage of health services [11], and the emerging infectious diseases result from a complexed interaction of socio-ecological factors will be regional challenges to control in these undeveloped settings [12]. The previous studies indicated that social and economic factors might contribute to transmission of infectious diseases such as dengue fever [13], and these factors play a significant role in predicting the changes in risk of infectious diseases caused by climate change [14,15]. Nevertheless, the research on climate-sensitive health outcomes, including infectious diseases has been scantly conducted in developing countries with subtropical and tropical climates [16–18].

Vietnam is a tropical and developing country and one of top 5 countries most vulnerable to climate change in the South-East Asia [19]. The annual average temperature in Vietnam increased approximately 0.5°C during the last 50 years (1958–2007) [20] and is projected to increase from 1.1 ^o^C to 3.6 ^o^C by the end of the 21^st^ century [21]. The heatwave events with the temperature of over 40°C and frequency of days with average temperature above 35 ^o^C have been recorded in a majority of areas, especially highly vulnerable areas such as the Mekong Delta region of Vietnam [22,23]. The heavy rainfalls have been found to increase up to 20% over the last 50 years in the Southern central region of Vietnam, and sea level rise along Vietnam coasts is at a rate of about 2.8 mm/year [20]. The previous studies [18,24–28] have revealed the association between extreme weather (i.e. high temperatures, heavy rainfalls and flooding) and elevated risk of hospitalization, water- and vector-borne diseases. However, these studies were limited to a sole disease (dengue, or diarrhoea) in a single city or region. Moreover, a little study had a look at the influences of the combination of climatic and socio-economic factors on temporal variation of communicable diseases at a large scale across the country.

This study aims to examine the effects of socio-ecological factors, which comprise climatic and socio-economic factors, on multiple communicable diseases at the national scale of Vietnam.

## Methods

### Data collection

We obtained monthly data on a number of notified cases of communicable diseases from Vietnam General Department of Preventive Medicine (VGDPM), Ministry of Health that is the leading agency responsible for prevention and control of communicable diseases at the national level [29]. In accordance to the Prevention and Control of Infectious Disease Law (PCIDL), practitioners in hospitals and clinics are requested to report cases of 28 notifiable diseases to provincial preventive medicine centres (PMCs) within 24 hours, and then PMCs reported number of cases of these diseases to VGDPM daily, weekly or monthly depending on types of diseases. We selected 9 diseases, which we could obtain sufficient number of cases for examining monthly variation, including diarrhoea, shigellosis, amebiasis, chickenpox, mumps, influenza, dengue, malaria, and rabies during the period from 2011 to 2015. These diseases are representative for the class of oral-, airborne-, vector-, and animal-transmission diseases. We collected weather data comprising temperature, humidity and cumulative rainfalls from 8 meteorological stations which were representative for 8 ecological regions (Red River Delta, North-East, North-West, North-Central, South-Central, South-East, Highland, and Mekong Delta region). The weather data of each region was then used for the individual province/city that located in the ecological region. The accessible socio-economic factors (population density, monthly average income, % illiteracy, % of households with supplied safe water, and number of the passengers by road) were collected from Vietnam General Statistics Office [30] and Vietnam Health Statistic Yearbook [31]. These indicators were chosen because they were available on the public website of Vietnam General Statistics Office, and they were revealed to have effect on the climate-disease relationship in elsewhere in Vietnam [18,32]. The ethic clearance was provided by Griffith University Human Research Ethics Committee (GUHREC).

### Data analysis

We first used *Global Moran’s I* to initially evaluate spatial clustering of each disease. *Moran’s I* is a measure of spatial autocorrelation which is characterized by a correlation in a signal among nearby locations in space. If significant spatial autocorrelation was found, we then used local indicators of spatial autocorrelation (*LISA*) to evaluate the location of disease clusters. Second, we used multilevel negative binomial regression model or zero-inflated negative binomial regression (for disease with an extensive number of zero values) using the Bayesian framework to analyse the association between socio-ecological factors and variation of each communicable disease. The spatial autoregressive models comprising spatial lags, which were a weighted average of observations on the diseases over neighbouring units, were input into the model to adjust for spatial variation of the disease outcomes. Modelled values of temperatures and humidity were centred on the mean values for each variable. The seasonal effect was controlled by dummy variables of the month of a year, and the population was controlled using its offset variable. The general model is described in Eq 1.

$$y_{tdi}=\alpha +\gamma \sum_{j=1}^{n}w_{ij}\;y_{j}+\beta_{1}T_{t}+\beta_{2}H_{t}+\beta_{3}R_{t}+\sum S_{n}+\sum M_{t}+\varepsilon_{i}$$

where, *y*_*i*_ denotes the monthly counts of Disease *d* in Province *i* at month *t*; $\sum_{j=1}^{n}w_{ij}{\;y}_{j}$ is a spatial lag, and the *w*_*ij*_ are the spatial weights; *S*_*n*_ is socio-economic factors: all variables were included at one time in Eq 1; *T*_*t*_ is the monthly temperature on month t; *H*_*t*_ is the monthly humidity; and *R*_*t*_ is monthly cumulative rainfall; *M*_*t*_ is the dummy variable of month t; and *ε*_*i*_ is a random intercept.

We conducted an initial burn-in of 2,500 iterations that were default subsequently discarded, and a subsequent set of 10,000 iterations was conducted for the model. The convergence was assessed by visual inspection of posterior density plots, history plots, and autocorrelation of selected parameters. Non-informative *N(0*,*1000)* priors were used for all means and Gamma(1,1) priors for variances [33]. Sensitivity analyses with maximum and minimum temperature as instead of average temperatures were also conducted using the same procedures, in which we also used the same non-informative priors for the minimum and maximum temperatures. We used the package “Bayes:” developed for Stata software version 15.0 to analyse data using the Bayesian framework (StataCorp. 2017. *Bayesian Analysis Reference Manual Release 15*. College Station, TX: Stata Press).

## Results

Table 1 presents the summary statistics of communicable diseases and socio-ecological variables. The means of monthly average temperatures varied by ecological regions and ranged from 22.2 to 28.2°C, and the means of humidity and cumulative rainfall were in a range of 74.3–81.2% and 0.4–3.5 mm respectively. The average monthly numbers of diseases are 852, 50, 27, 45, 34, 1605, 93, 42, and 429 for diarrhoea, shigellosis, amebiasis, chickenpox, mumps, influenza, dengue, malaria, and rabies respectively.

**Table 1:** Descriptive statistics socio-ecological factors and communicable diseases.

**Socio-ecological Variables**	**Minimum**	**25**^**th**^	**50**^**th**^	**Mean**	**75**^**th**^	**Maximum**
Climatic factors by ecological regions: Temperature (^o^C), Humidity (%), Rainfall (mm)						
Red River Delta	12.3, 60.2, 0	19.5, 73, 0	25.1, 76.7, 0	24.2, 77.0, 0.4	28.9, 81.9, 0	30.4, 88.3, 7
North East	12.4, 60.2, 0	19.5, 73, 0	25.1, 76.7, 0	24.2, 77, 0.4	28.9, 81.9, 0	30.4, 88.3, 7
North West	12.2, 59, 0	18.9, 69.5, 0.3	23.5, 75.2, 1.7	22.2, 74.3, 2.2	25.6, 79, 3	27.5, 84, 8
North Central Coast	18.1, 56.6, 0	23.2, 73.9, 0.03	26.0, 84.6, 1.3	25.8, 81.2, 2.7	29.2, 87.7, 3.96	31.3, 90, 27
South Central Coast	19.1, 63.2, 0	24.2, 71.1, 0.3	26.7, 78.2, 1.5	26.3, 77.1, 3.5	29.8, 81.1, 4.7	31.3, 90, 27
Highland	19.1, 59.8, 0	22.9, 70.4, 0	25.6, 77.9, 0.6,	25.6, 75.9, 2.9	28.9, 81, 3.3	31.1, 90, 27
North East of the South	25.2, 60.2, 0	27.4, 71.2, 0	28.3, 75.9, 0	28.2, 76.3, 0.5	28.9, 82.7, 0	30.8, 89.3, 5.4
Mekong Delta (West of the South)	25.6, 62.9, 0	27.8, 70.3, 0	28.2, 76.1, 2.6	28.2, 74.7, 3.2	28.8, 78.9, 5.9	30.1, 84.9, 11.5
Socio-economic factors						
Population density (* 1000 persons/km^2^)	0.05	0.13	0.27	0.48	0.67	3.73
Monthly average income per capita (*1000 VND)	987	1673	2174	2268	2642	4839
% of Illiteracy	2.4	3.5	6.4	8.9	9.3	42.6
% of households with safe water supply	17.2	68.8	88.2	79.8	96.7	96.7
Number of passengers by road (*1000 persons)	0.02	0.39	0.85	1.42	1.42	13.1
Communicable Diseases (average monthly counts)						
Diarrhoea	0	381	623	852	947	15876
Shigellosis	0	7	25	50	65	1071
Amebiasis	0	2	11	27	34	912
Chicken pox	0	11	25	45	56	710
Mumps	0	7	19	34	41	564
Influenza	0	468	1216	1605	2052	14715
Dengue	0	0	11	93	82	5610
Malaria	0	2	14	42	57	942
Rabies	0	82	216	429	566	6277

**Fig 1:**
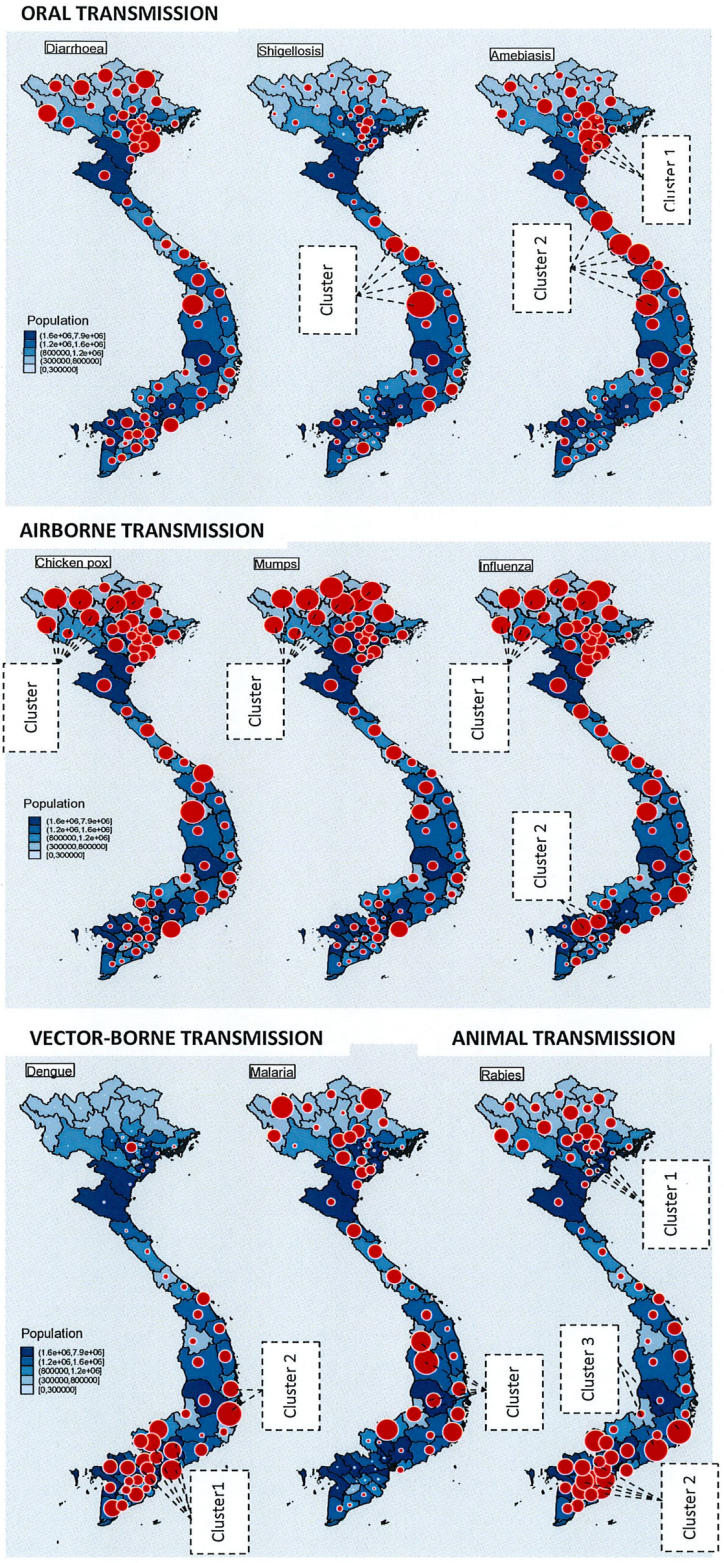
Spatial distribution of incidence of each disease.

Fig 1 shows the spatial distribution of monthly incidence rates of each disease by provinces. The monthly incidence of diarrhoea ranged from 8.8 to 357.4 per 100,000, and the higher incidences were found in the mountainous provinces locates in the North-West region. However, no provincial cluster of diarrhoea was found (Moran’s I, 0.06; p-value, 0.2). The average monthly incidence of shigellosis ranged from 0.05 to 54.7 per 100,000, and the higher incidences were found in Central regions with a significant high-high risk cluster comprising Quang Tri, Thua Thien Hue, Kontum and Quang Nam province (Moran’s Li, 1.4–2.7; p-value<0.05). The average monthly incidence of amebiasis ranged from 0.0009 to 9.5 per 100,000, and higher incidences were observed in the Red River Delta and Central regions. Two significant clusters were found in these regions, comprising Thai Binh, Ha Nam, Hung Yen (Cluster 1, Moran’s Li, 0.9–2.1, p-value<0.05), and Quang Binh, Quang Tri, Hue, Quang Nam, Kontum province (Moran’s Li, 1.9–5.8, p-value<0.05). The monthly incidences of chickenpox ranged from 0.25 to 15 per 100,000, and higher incidences were found in the North-West region. A significant cluster was formed by Dien Bien, Tuyen Quang, Bac Kan, Yen Bai, Lao Cai, and Lai Chau province (Cluster 1, Moran’s Li, 1.2–3.5, p-value<0.05). The monthly incidences of mumps ranged from 0.23 to 13.7 per 100,000, and higher incidences were found in North-West region with a significant cluster formed by Dien Bien, Lang Son, Yen Bai, Lai Chau, Tuyen Quang, Lao Cai, Bac Kan, Cao Bang, and Ha Giang province (Moran’s Li, 1.4–5.4, p-value<0.05). The monthly incidences of influenza ranged from 0.3 to 491 per 100,000, and higher incidences were also found mostly in North-West region. Two significant clusters were observed in North-West (Dien Bien, Ha Giang, Bac Kan, Lao Cai, Lai Chau, Cao Bang; Moran’s Li, 2.1–3.5, p-value<0.05) and the South-East (Ho Chi Minh City and Dong Nai; Moran’s Li, 0.73, p-value<0.05). The monthly incidences of Dengue ranged from 0 to 32.9 per 100,000, and the high incidences were mostly found in Southern provinces. Two significant clusters were forms by Ho Chi Minh city, Long An, Tay Ninh, Binh Phuoc, Binh Duong, Dong Nai, Ba Ria Vung Tau (Cluster 1, Moran’s Li, 0.9–2.6, p-value<0.05), and Phu Yen and Khanh Hoa province (Cluster 2, Moran’s Li, 0.8 & 1.6, p-value< = 0.05). The monthly incidences of malaria ranged from 0.02 to 23 per 100,000, and one significant cluster was found in Highland region, comprising Dak Nong, Dak Lak, Gia Lai, and Kontum (Moran’s Li, 1–1.9, p-value<0.05). The monthly incidences of rabies ranged from 1.7 to 117.2 per 100,000 with higher incidences observed in Red River, South Central, and the Mekong Delta regions. Three significant clusters were found, including Nam Dinh, Hai Phong, Hai Duong, Hung Yen, Ha Nam and Thai Binh (Cluster 1, Moran’s Li, 0.7–1.2, p-value<0.05), and Dong Thap, Tien Giang, Can Tho, Ben Tre, Tra Vinh, Vinh Long province (Cluster 2, Moran’s Li, 0.8–2.7, p-value<0.05), and Ninh Thuan, Binh Thuan province (Cluster 3, Moran’s Li, 1.8 & 2.3, p-value<0.05).

The associations between socio-ecological factors and risk of diseases are presented in Table 2. A decree increase in the mean of average temperature was significantly associated with 0.4% (95CI, 0.3–0.5), 2.5% (95%CI, 1.4–3.6), 0.9% (95%CI, 0.6–1.4), 1.1% (95%CI), 5% (95%CI, 3-.7.4), 0.4% (95%CI, 0.2–0.7), and 2% (95%CI, 1.5–2.8) in incidence of diarrhoea, shigellosis, mumps, influenza, dengue, malaria, and rabies respectively. However, none-statistically significances were found in the relationship between temperatures and amebiasis and chickenpox. The sensitivity analyses (Table A in S1 File) reflect that temperature has a V-shape relationship with influenza (1 decree decrease in minimum temperature associated with 0.07% increase in incidence of influenza), and the cold weather is significantly associated with chickenpox (a decree decrease in minimum temperature associated with 1.4% increase in incidence of chickenpox). The average humidity had a significantly positive association with diarrhoea (0.3%; 95%CI, 0.1–0.5), shigellosis (0.8%; 95%CI, 0.4–1.1), amebiasis (1.7%; 95%CI, 1.3–2.0), mumps (0.4%; 95%CI, 0.001–0.9), influenza (1.2%; 95%CI, 0.9–1.5) and a significantly negative association with dengue (-3.7%; 95%CI, -3.7-(-2.4) and rabies (-0.5%; 95%CI, -0.9-(-0.2). Cumulative rainfall positively associated with increase in incidences of shigellosis (3.1%; 95%CI, 2–4.2), dengue (15%; 95%CI, 13.1–17), and rabies (3.5%; 95%CI, 3.2–3.7) but it negatively associated with diarrhoea (-0.3%; 95%CI, -0.4-(-0.2)), amebiasis (-0.5%; 95%CI, -0.8-(-0.2)), chickenpox (-1.3%; 95%CI, -2.3-(-0.8)), mumps (-1.8%; 95%CI, -2.2-(-1.5)), influenza (-0.2%; 95%CI, -0.3-(-0.1)), and malaria (-2.1%; 95%CI, -2.3-(-1.9)). In terms of socio-economic factors, increases in population density was a highly sensitive factor for elevated risk of diarrhoea (97%; 95%CI; 97.5–97.7), influenza (17%; 95%CI, 16.3–17.9), and rabies (17%; 95%CI, 16.6–17.9), but it is noteworthy that high population density was association with significant reduction of malaria (-26.9%; 95%CI, -27.2-(-26.4)). This might be due to the reversed association between urbanization and malaria. The monthly average incomes were found to be associated with subtle reductions of diarrhoeal diseases (diarrhoea, -0.04%, 95%CI, -0.05-(-0.02)) but a small elevation of chickenpox and dengue (0.04%, 95%CI, 0.02–0.05; and 0.1%, 95%CI, 0.09–0.1, respectively). The percentages of illiteracy significantly associated with increases of 0.6% (95%CI, 0.5–0.8), 3.8% (95%CI, 3.0–4.8), 17.9% (95%CI, 15.4–20.1), 0.7% (95%CI, 0.5–0.8), and 1% (95%CI, 0.6–1.5) of diarrhoea, shigellosis, dengue fever, malaria and rabies respectively. The percentages of households with supplied safe water were found negatively associated with diarrhoea, chickenpox and mumps but positively associated with shigellosis, amebiasis, and dengue (Table 2). The increase (*1000) in number of the passengers by road had a significant positive relationship with influenza (3.9%; 95%CI, 3.3–4.4) and dengue (11.9%; 95%CI, 10.1–13.4) however it had a reversed association with malaria (-4.8%; 95%CI, -5.2-(-4.4). The results of convergence diagnostics reflect the high proportion of proposed parameter values that were accepted by the models (Acceptance rates ranged from 32 to 38%). The visual inspection revealed that the levels of convergence were varied by variables. Examples of visual plots of convergence diagnostics for the parameter of average temperature in relation to the outcomes are presented in Figure A in S1 File.

**Table 2:** % change & 95% credible interval.

**Socio-ecological Variables**	**Oral Transmission**			**Airborne Transmission**			**Vector-Borne & Parasite Transmission**			**Animal Transmission**
	Diarrhoea	Shigellosis	Amebiasis	Chickenpox	Mumps	Influenza	Dengue	Malaria		Rabies
**Climatic Factors**										
Average Temperature (^o^C)	0.4* (0.3–0.5)	2.5* (1.4–3.6)	-0.3 (-1.5–0.7)	0.08 (-0.03–0.3)	0.9* (0.6–1.4)	1.1* (0.7–1.5)	5* (3–7.4)		0.4* (0.2–0.7)	2.0* (1.5–2.8)
Average Humidity (%)	0.3* (0.1–0.5)	0.8* (0.4–1.1)	1.7* (1.3–2.0)	-0.3 (-0.6–0.3)	0.4* (0.001–0.9)	1.2* (0.9–1.5)	-3.1* (-3.7-(-2.4)		0.2 (-0.1–0.5)	-0.5* (-0.9-(-0.2)
Cumulative Rainfall (mm)	-0.3* (-0.4-(-0.2)	3.1* (2.0–4.2)	-0.5* (-0.8-(-0.2)	-1.3* (-2.3-(-0.8)	-1.8* (-2.2-(-1.5)	-0.2* (-0.3-(-0.1)	15* (13.1–17.0		-2.1* (-2.3-(-1.9)	3.5* (3.2–3.7)
**Socio-economic Factors**										
Population density (* 1000 persons/km^2^)	97.6* (97.5–97.7)	-	-	8.4* (8.2–8.6)	-4.0* (-4.4-(-3.4)	17* (16.3–17.9)	-		-26.9* (-27.2-(-26.4)	17* (16.6–17.9)
Monthly average income per capita (*1000 VND)	-0.04* (-0.05-(-0.02)	-0.009 (-0.008-(-0.003)	-0.04* (-0.05-(-0.03)	0.04* (0.02–0.05)	0.007 (-0.008–0.02)	-0.06 (-0.07–0.05)	0.1* (0.09–0.1)		0.001 (-0.01–0.01)	0.003 (-0.01–0.01)
% of Illiteracy	0.6* (0.5–0.8)	3.8* (3.0–4.8)	-0.2 (-0.9–0.6)	0.09 (-0.6–0.4)	-1.6* (-1.9-(-1.2)	-0.07 (-1.2–1.0)	17.9* (15.4–20.1)		0.7* (0.5–0.8)	1.0* (0.6–1.5)
% of households with safe water	-0.1 (-0.3–0.1)	2.0* (1.7–2.3)	2.1* (1.7–2.4)	-1.2* (-1.7-(-0.7)	-0.4* (-0.5-(-0.2)	-	0.9* (0.5–1.2)		-	-
Number of passengers by road (*1000 persons)	-	-	-	-	-	3.9* (3.3–4.4)	11.9* (10.1–13.4)		-4.8* (-5.2-(-4.4)	-

*Statistically significant at p-value< = 0.05.

## Discussion

This is the first study—examining the relationships between socio-ecological factors and multiple communicable diseases across Vietnam. The study applied the multi-level negative binomial combined with the spatial autoregressive model using a Bayesian framework for data analyses. The results indicated that all of diseases, comprising diarrhoea, shigellosis, amebiasis, chickenpox, mumps, influenza, dengue, malaria, and rabies, were associated with at least 2 out of 3 climatic factors (temperatures, humidity, and cumulative rainfall). Dengue incidence was found to be the most sensitive with climatic factors. The influences of socio-economic factors on risk of selected communicable diseases are varied by factors, and the biggest contribution to changes in risk of diseases was found with the population density.

The observed positive association between temperatures and risk of diarrhoea in this study is consistent with the findings reported from previous studies. A systematic review by Levy et al [34] indicated that 69% of analyses of all-cause diarrhoea and 79% of analyses of bacterial pathogens found a significant positive association between temperature and diarrhoea. The previous studies by Phung et al [26] indicated that a 1°C increase in temperature at lag one month was associated with 1.1% in risk of all-cause diarrhoea [35], and high relative humidity was associated with 13% in risk of all-cause diarrhoea in the Mekong Delta region [26]. The plausible mechanisms on the temperature-diarrhoea relationship may be explained by several ways. First, temperatures directly influenced on replication and survival of pathogens [36]. Second, high temperatures lead to an increase and rapid pathogen growth in foods, resulting in increased risk of food poisoning [37]. Third, changes in temperatures cause in a variation of dietary patterns and hygiene behaviour. For instance, higher consumption of water during hot weather and reduced of hot, cooked foods facilitate transmission of bacteria and other pathogens [38]. In the contrary, the positive association between humidity risk of diarrhoea found in this study is inconsistent with the negative effects of humidity on diarrhoea observed in previous studies [39,40]. However, the study illustrated that humidity alone did not contribute significant influence on diarrhoea but under the interaction with temperatures and rainfall [40]. The positive association between cumulative rainfall and elevated risk of shigellosis observed in this study was also revealed in the highland provinces in the previous studies [41,42], however the inversed relationship between rainfall and all-cause diarrhoea reflects inconsistent evidence on the effects of rainfall to diarrhoea. The review by Levy et al [34] showed that location-specific studies of an association between heavy rainfall and diarrhoea have found mixed results. These might be due to geographical features (e.g. elevation)[43] and seasonal sequence of rainfall (i.e. after dry or after wet season) [34]. Further study for better understanding of the interaction between geographic and seasonal patterns with climatic factors in relation to risk of diarrhoea should be conducted.

For airborne transmission diseases, the influence of temperature and humidity on mumps found in this study was also reported from the previous studies. Onozuka et al [44] and Ho et al [45] indicated that a 1°C increase in temperature associated with 3.9% and 7.5% increase in mumps incidence, and 1% increase in relative humidity associated with 1.4% increased mumps incidence [44,45]. The mechanism of temperature and humidity in the transmission of mumps virus is still unclear however the potential explanation can be due to increase in physical activity and contacts with children during warmer weather that could, in turn, promote mumps infection [44]. This may be used to explain for a negative association between rainfall and mumps found in this study due to less activity during wet days. The positive temperature-influenza relationships found in this study was in agreement with the previous studies conducted in sub-tropical and tropical countries such as Vietnam [46], Philippines [47], and South America [48]. Similarity, the positive correlation was also reported for the relationship between absolute humidity and influenza [46]. The underlying mechanism for these phenomena might be explained by the variability of influenza virus in airborne respiratory droplets [49,50]. During high humid condition, low evaporation of respiratory droplets allows the virus to maintain its viability in the environment [51]. These droplets then remain onto surfaces owing to gravity, then creating a reservoir for contact transmission [50]. Moreover, humid conditions were previously revealed to be instead conductive for contact transmission in tropical settings [51,52].

For vector-borne transmission diseases, dengue was the most sensitive to climatic factors. In this study, dengue incidence was found strongly associated with temperatures and rainfalls. These findings were strongly agreement with previous studies in Vietnam [27,42,53]. Temperature has been revealed to play important role in biting rate, egg and immature mosquito development, developmental time of virus in mosquito, and survival at all states of the mosquito life cycle [54], and experimental study [55] indicated that temperature range for survival through all life phases of *Ae*. *aegypty* is between 20 and 30°C. Similarly, an increase in precipitation facilitates habitats for aquatic stages of the mosquito life and strongly influences vector distribution [56]. The study in the relationship between humidity and dengue have received less attention, and humidity is likely influencing on vector competence under the interaction with temperatures but not alone [57]. The positive association between temperature and malaria observed in this study was also reported from previous studies [58,59]. However, the negative relationship between rainfall and malaria found in this study is inconsistent with that observed in the previous study. The answer for this inverted relationship is unclear however this might be due to the highly variation of the relationships according to the season and geography. For instance, the study by Briet et al [60] in Sri Lanka revealed that inter-annual analysis found strong negative correlations between malaria and rainfall. Further study in a climate-malaria relationship under the contexts of regional climate and interaction of climatic factors and sociodemographic factors should be conducted for a better understanding of this relationship mechanism.

The association between weather factors and human rabies has been received more attention recently. Our study found a positive association between temperature and incidence of rabies which reflects consistence in evidence reported from other studies [61,62]. For instance, Yao et al (2015) reported an increase of 19% of rabies corresponding to a 5°C increase in temperature at lag 1 month. The positive high temperature–rabies relationship may be attributed to the wearing less and having more outdoor activities (in the absence of indoor air-conditioning in rural areas) during the hot weather, thus people have more frequent contact with carnies, irascibility of which is sensitive to high temperatures [61]. The positive association between monthly rainfalls and rabies found in this study supported for the evidence observed in the study conducted by Rifakis et al [63] which indicated that most cases of rabies occurred in a lowland area and during the rainy season. The study suggested the change from El Niño to La Niña might affect many diseases, including rabies. However, the plausible mechanism of this relationship is still unclear.

In terms of socio-economic factors, population density was found to be an important factor influencing the variation of diseases, especially with diarrhoea, chickenpox, influenza, and rabies. However the population density was negatively associated with malaria. This may relate to the interaction between population density and urbanization. The study conducted by Kabaria et al [64] indicated that the risk of malaria infection was shown to decline from rural areas through peri-urban settlements to urban central areas. Likewise, the influences of other socio-economic factors are varied and inconsistent by different diseases. For instance, income was found slightly associated with a reduction in oral transmission diseases and influenza but not related to vector-borne and animal transmission diseases. The previous study [57] expressed that in addition to climatic factors, socioeconomic factors and public health determinants also play an important role in driving spatial patterns of vectors such as dengue transmission. However, the interaction between these factors, weather and other environmental factors are very complex and vary spatially and temporally, and can result in non-linear feedback. Future studies with more sophisticated designs should be conducted to better understanding these complex interactions.

This study faces on some limitations. First, the time-span of data was limited in 5 years only due to an availability of data collected from the surveillance system, so changes in area-level factors such as some socio-economical characteristics may not occur in a short period of time. This might reduce the statistical power to detect the relationship between these factors and variation of diseases. Second, the data provided by the surveillance reports did not include cause-specific diarrhoeal diseases, so this study could not examine the relationship between socioecological factors and cause-specific diarrhoeal disease. Finally, as an ecological study design, this study had no access to individual-level data such as information on adaptive measures (e.g. air conditioning), occupation, or outdoor/indoor dynamic activities which may reflect the actual exposure to temperatures of each individual. It is hard to deal with this limitation due to complication monitoring of temperature exposures for each individual, however, the future study can use a hybrid study design which use individual-level data of a sub-population to improve ecological inference.

## Conclusion

Our study provides the description of the spatial distribution of multiple communicable diseases and the time-series association between these diseases and socioecological factors at the national scale of Vietnam. The study found that oral transmission diseases (i.e. diarrhoea) were spatially distributed across the country; whereas, the airborne transmission diseases (chickenpox, mumps, and influenza) are more likely clustered in the North-Western region, and vector-borne transmission diseases (dengue, malaria) were more likely clustered in the Southern regions. Climatic factors were found to be sensitive to the temporal variation of the diseases, especially temperature, rainfall and dengue fever. The population density was strongly associated with risk of communicable diseases while other socio-economic factors were weakly and inconsistently associated with the risk of communicable diseases in this study. Our findings can help better understanding of how the diseases spatially distribute and how the socioecological factors affect the transmission of communicable disease at the national scale which has never been done before in Vietnam. In addition, the relationship between climatic factors and communicable diseases reflects an important implication for the climate change adaptation strategies and public health decision, of which development of weather-based early warning systems should be considered to strengthen communicable disease prevention and control.

## Supporting information

S1 File Supplemental information on sensitivity analysis, convergence diagnostic, and the map of ecological region in Vietnam. (PDF)
