# Supplementary material for: The effects of socioecological factors on variation of communicable diseases: A multiple-disease study at the national scale of Vietnam
Source: PLoS One. 2018 Mar 1;13(3):e0193246. doi: 10.1371/journal.pone.0193246 (PMC5832231; doi:10.1371/journal.pone.0193246)

S1 File: Supplemental information on sensitivity analysis, convergence diagnostic, and the map of ecological region in Vietnam

Table A. Sensitivity Analysis with Maximum and Minimum Temperatures

	Oral Transmission			Airborne Transmission			Vector-Borne & Parasite Transmission		Animal Transmission
	Diarrhoea	Shigellosis	Amebiasis	Chicken pox	Mumps	Influenza	Dengue	Malaria	Rabies
Maximum Temperature (^{o}C)	0.6 (0.46-0.85)	2.1 (1.24-3.0)	-0.000 (-0.9-0.98)	1.2 (1.1-1.3)	1.2 (0.7-2.1)	1.6 (1.5-1.7)	-1.8 (-3.7-0.3)	1.5 (1.2-1.9)	1 (0.3-1.4)
Minimum Temperature (^{o}C)	0.5 (0.29-0.57)	2.0 (1.0-3.0)	-0.6 (-1.6-0.5)	-1.4** (-1.8-(-1.0)	0.4 (0.1-0.7)	-0.07** (-0.1-(-0.04)	11.4 (9.9-12.9)	-1.0* (-1.3-(-0.7)	0.9 (0.4-1.5)

** Different from average temperature

Figure A. Examples of convergence diagnostics of MCMC for each disease and centred average temperature

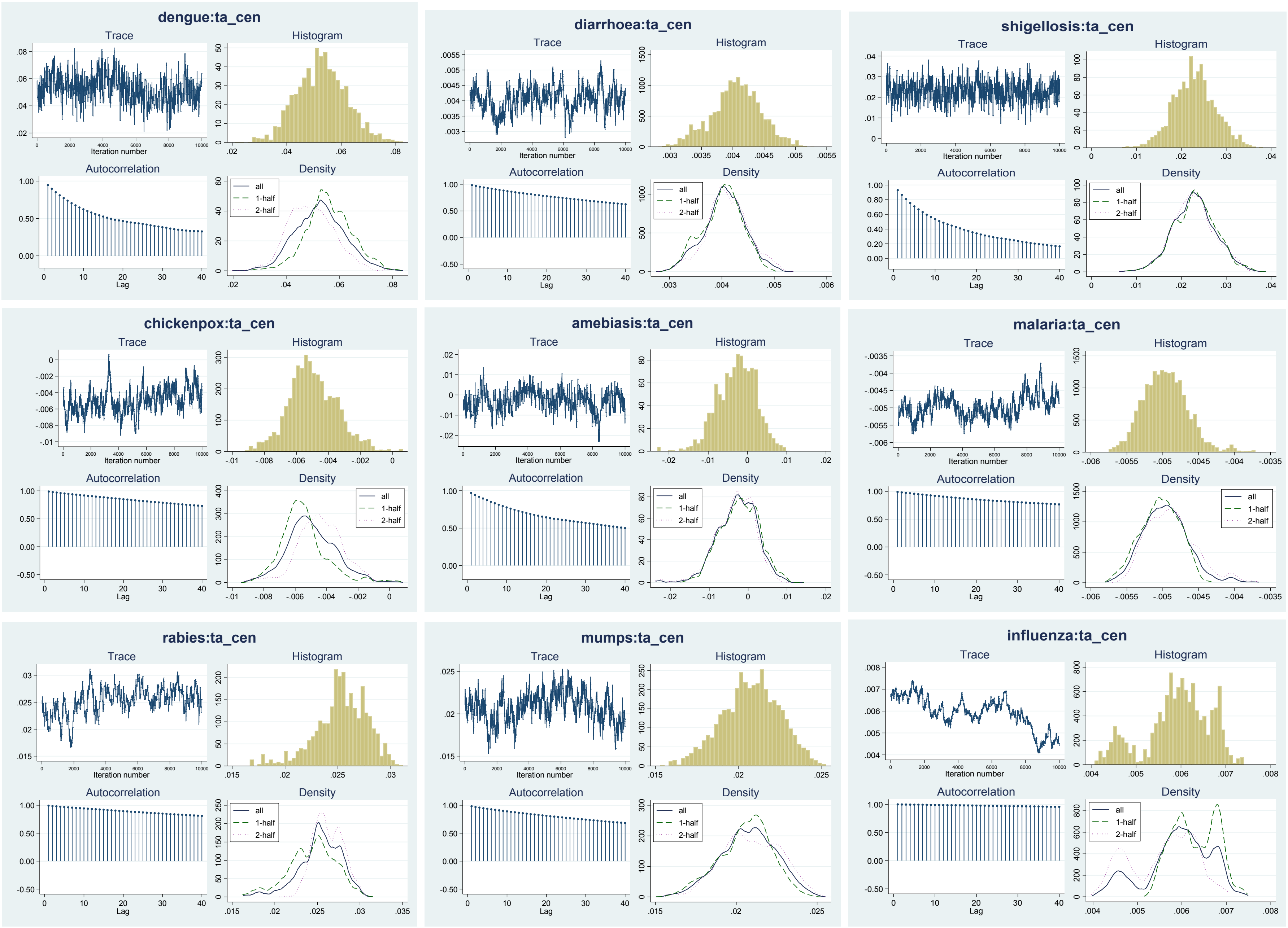

Figure B. Map of ecological regions in Vietnam (Source: www.threeland.com)

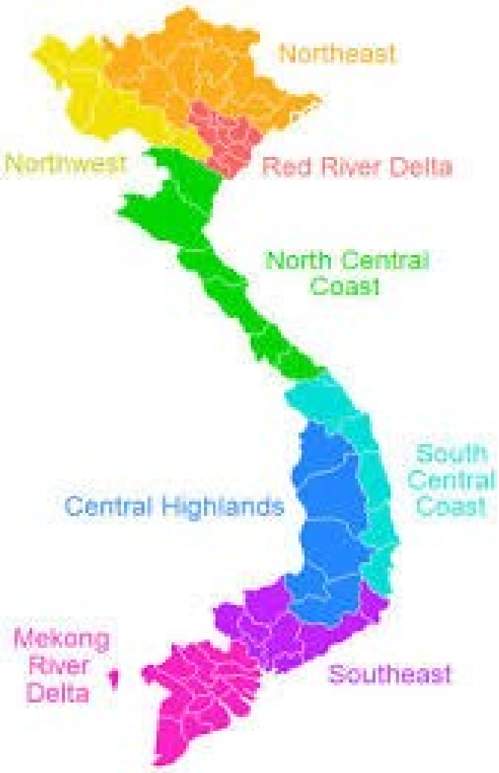

Supplement: S1 File — (PDF) [file pone.0193246.s001.pdf]
